# Comparative transcriptomic analysis of the tea plant (*Camellia sinensis*) reveals key genes involved in pistil deletion

**DOI:** 10.1186/s41065-020-00153-x

**Published:** 2020-09-08

**Authors:** Yufei Liu, Dandan Pang, Yiping Tian, Youyong Li, Huibing Jiang, Yunnan Sun, Lifei Xia, Linbo Chen

**Affiliations:** 1 Tea Research Institute, Yunnan Academy of Agricultural Sciences, Menghai, 666201 China; 2 Yunnan Provincial Key Laboratory of Tea Science, Menghai, 666201 China

**Keywords:** *Camellia sinensis*, Special germplasm, Pistil deletion, ABCDE model, Ethylene

## Abstract

**Background:**

The growth process of the tea plant (*Camellia sinensis*) includes vegetative growth and reproductive growth. The reproductive growth period is relatively long (approximately 1.5 years), during which a large number of nutrients are consumed, resulting in reduced tea yield and quality, accelerated aging, and shortened economic life of the tea plant. The formation of unisexual and sterile flowers can weaken the reproductive growth process of the tea plant. To further clarify the molecular mechanisms of pistil deletion in the tea plant, we investigated the transcriptome profiles in the pistil-deficient tea plant (CRQS), wild tea plant (WT), and cultivated tea plant (CT) by using RNA-Seq.

**Results:**

A total of 3683 differentially expressed genes were observed between CRQS and WT flower buds, with 2064 upregulated and 1619 downregulated in the CRQS flower buds. These genes were mainly involved in the regulation of molecular function and biological processes. Ethylene synthesis–related ACC synthase genes were significantly upregulated and ACC oxidase genes were significantly downregulated. Further analysis revealed that one of the WIP transcription factors involved in ethylene synthesis was significantly upregulated. Moreover, AP1 and STK, genes related to flower development, were significantly upregulated and downregulated, respectively.

**Conclusions:**

The transcriptome analysis indicated that the formation of flower buds with pistil deletion is a complex biological process. Our study identified ethylene synthesis, transcription factor WIP, and A and D-class genes, which warrant further investigation to understand the cause of pistil deletion in flower bud formation.

## Introduction

Tea plant [*Camellia sinensis* (L.) O. Kuntze], originating from the southwest region of China [1], is a crucial perennial evergreen leafy plant and economically valuable woody crop. The tender shoots are harvested from cultivated tea plants, and the flourishing growth of branches and leaves of the tea plant are the prerequisites for high yield and stable production of tea gardens [2]. Therefore, flower bud differentiation to tea fruit maturation requires 1.5 years, during which time, the plant consumes large quantities of nutrients, resulting in a decline in tea yield and quality, and accelerated aging of the tea plant [3, 4]. The research on tea flowers mainly focused on hybridization and incompatibility in the tea plant [5–11]. However, the research on unisexual flowers and parthenogenesis of tea plant are very little. The current study demonstrated that the formation of unisexual flowers in the tea plant could effectively reduce their reproductive growth [4]. Therefore, studying the flower development mechanism could reveal new information for regulating the sex determination of the tea plant and other species.

The sex differences between flowering plants are mainly related to flower organs [12, 13]. Therefore, the genes associated with flower development may be involved in the sex differentiation process. According to the ABCDE model of flower development, genes of the B + C + E class regulate stamen development, those of the C + E class regulate carpel development, and those of the D class regulate ovule development [14–17]. The genes of the B, C, D, and E classes synergistically regulate the development of stamens, carpels, and ovules [18]. The vast majority of functional genes involved in the ABCDE model belong to the MADS-box gene family, which indicates that this gene family regulates flower sex differentiation [14, 16, 19]. For example, weak mutants and RANi strain of the AG gene cause carpel development to be deficient and the flower meristem to lose certainty; however, the development of the stamen is almost unaffected [20, 21].

Furthermore, various plant hormones are involved in the sex determination of plants [22, 23]. Among them, the gaseous hormone ethylene plays a large role [24–26]. Methionine adenosyltransferase, ACC synthase (ACS), and ACC oxidase (ACO) are the enzymes required for ethylene biosynthesis, of which ACS and ACO are key enzymes for ethylene synthesis [27–29]. Studies have indicated that high endogenous ethylene concentrations and elevated expressions of the *CsACS1* [30], *CsACS2* [31], *CsACS11* [32], *CmACS7* [33], *CmACS11* [34], and *CsACO2* [35] genes are associated with the formation of female flowers in cucumbers (Cs) and melons (Cm). In melons, *CmWIP1*, a C2H2 zinc finger transcription factor gene, indirectly downregulates the expression of *CmACS7*, thereby promoting the formation of male flowers. Furthermore, a loss-of-function mutation in the *CmWIP1* gene induces the formation of gynoecious plants [34]. The ortholog CsWIP1 of CmWIP1 can bind to the promoter of *CsACO2* and suppress its expression, thereby regulating the development of unisexual flowers in cucumbers [35]; *ClWIP1* was determined to be involved in the sex determination of watermelons [36].

The tea plant, similar to most angiosperms, has bisexual flowers. Distinguishing the sex of the tea plant with bisexual flowers is impossible, which limits the study of sex determination of the tea plant. In the early stages of 2016, our research group identified a natural pistil-deficient tea plant (CRQS) [37]. The flowers of CRQS were unisexual (male) and did not exhibit styles or ovaries [37]. The characteristics of CRQS provide valuable insights for further study of pistil deletion and sex determination in the tea plant. To determine the molecular mechanism of pistil deletion in tea plants, we profiled high-throughput RNA-Seq analysis of the flower buds of the CRQS, wild tea plant (WT), and cultivated tea plant (CT) to investigate the differentially expressed genes (DEGs). Furthermore, DEG expression was verified using real-time PCR, to clarify the key genes promoting CRQS pistil deletion. Our results provide a foundation for future studies on the sex determination of the tea plant and contribute to the selection of pistil-deficient tea plant varieties.

## Methods

### Plant materials

CRQS (*C. sinensis* vs. *assamica*), WT (*C. taliensis*), and CT (*C. sinensis* vs. *assamica*) were grown in the national germplasm repository of large-leaf tea (Menghai) of the Tea Research Institute, Yunnan Academy of Agricultural Sciences. A rare tea plant with pistil deletion, CRQS, with a natural unisexual flowers mutant was discovered [37]. The flower buds were separately collected from three tea plants on May 20, 2018. All the samples (flower buds) were immediately frozen in liquid nitrogen and stored at − 80 °C until further use. Three biological replicates were performed for all the experiments.

### cDNA library construction and sequencing

Total RNAs of the collected flower buds were extracted using previously described methods [38]. After preparation, the quality and quantity of RNA were measured using the Nanodrop 2500 (Thermo Fisher Scientific, USA) and agarose gel electrophoresis. The total RNAs of the samples were used to construct the RNA-Seq libraries. The cDNA libraries were constructed and transcriptome sequenced by Beijing Novogene Technology (Beijing, China) using the Illumina HiSeq 2500 platform. Chen et al. described the procedure in further detail [38].

### Genome alignment and gene annotation

The original image data files of the samples obtained from the Illumina HiSeq 2500 were converted to raw sequence reads through base calling analysis. The raw reads were filtered by removing adapter sequences and low-quality reads, and the raw sequence reads were converted to clean reads. The Hierarchical Indexing for Spliced Alignment of Transcripts (HISAT2) program was used to map the remaining reads to the tea reference genome (http://www.plantkingdomgdb.com/tea_tree/). Newly discovered genes that were not annotated in the reference genome were annotated using publicly available databases according to the methods described by Ma et al. [5]. The transcriptome data of this study was stored in the NCBI SRA database (SRA accession: PRJNA637178).

### Identification of DEGs

Transcript levels were calculated in fragments per kilobase of transcript per million mapped reads (FPKM). The DEGs between the two samples were defined as those exhibiting at least two-fold change in transcript abundance (|log2 (fold change)| ≥ 1), with *P* < 0.05 relative to a control, as obtained using the DESeq2 tool. Furthermore, at least one of the three samples had an FPKM > 1. The *P* values were adjusted using the Benjamin and Hochberg’s approach for controlling the false discovery rate [39].

### Functional annotation of transcripts

The nonredundant transcripts were annotated based on the following databases: Clusters of Orthologous Groups of proteins, euKaryotic Ortholog Groups, NCBI nonredundant protein sequences, homologous protein family, Gene Ontology (GO), and Kyoto Encyclopedia of Genes and Genomes (KEGG). A GO enrichment analysis of the DEGs was performed using the GOseq R package [40], and enriched pathways of DEGs were identified using KEGG Orthology Based Annotation System software.

### Quantitative real-time PCR verification

The first-strand cDNA was synthesized from 200 ng of total RNA using the FastKing gDNA Dispelling RT SuperMix (TIANGEN BIOTECH, China) according to the instruction manual. The transcripts or gene-specific primers used for qRT-PCR were designed with Primer Premier 5 software and synthesized using Sangon Biotech (Shanghai China). A qRT-PCR was performed using the KAPA SYBR® FAST qPCR Master Mi (2×) Kit (KAPABIOSYSTEMS, USA). The qPCR reactions were performed using the qTOWER 2.2 Real-time PCR System with the KAPA SYBR® FAST qPCR Master Mix (2×) Kit (KAPABIOSYSTEMS, USA). GAPDH (XM_028237220.1) from the tea plants was selected as a reference gene, and the relative expression level of each gene was calculated using the 2^-ΔΔCT^ method [41]. All qRT-PCR reactions were performed in triplicate, and the average Ct values were calculated. The primers used in this study are presented in Table 1.

**Table 1:** Primers used for qRT-PCR verification

Gene	Forward 5′-3’	Reverse 5′-3’
CSA026751	CCCCCAGTGAGGCTCTCTTT	TTGAAAGATGGCAAGCCACG
Novel05490	CGTGGATCGATAGGGCAAGC	TCGGAAACCCTACCCAATCC
Novel11742	CCATGCTGACAGTGGCTTCT	ATGTATAGACTTGCCTGCAATAGA
CSA010549	GCTTCTGGACTTGCTATGCG	CTTGATGAGCTCTGGACGGG
Novel02945	AGAAGGTGGCAGAGCTGTTG	TGTCAAGTTGGGTTGGGGAC
Novel00816	ACCAAGTGATCTTCCTGGTTT	AGGCATGCTATATTGAGGGGC
CSA026801	GAAACGATCCAATGCCTGCC	AGGCAATCCTATGTGCAGGG
CSA009488	GAGGCTCGAATTGGCAGGAT	GGTCAAAGTTGGTAACGGCG
Novel05812	GGTCTAACAATGGCCCAAGCAC	GCCTTCCCTAATGCTCTTTCCTCA
CSA014619	AAAGCTCGCGAACTCTCCAT	GTTAACGCAGAGACCGAGGA
Novel05521	GCACTAGCAATCAACAAGTGACC	GCTGCGCTCTCTGCGAAATA
CSA003190	TTCCAATCCATGCCCCACTC	TAAACAGTCCAGGCCTTCGC
CSA016398	CTCACGGTGAACCCTCCTTC	CTCGCATCGAATTGCAGAGC
Novel01833	AAGGATTTGTGTTGCCCTCG	ACAAGCTTGGCTTGCATGAG
GAPDH	TTGGCATCGTTGAGGGTCT	CAGTGGGAACACGGAAAGC

## Results

### Morphological characteristics of pistil-deficient flower buds

**Fig. 1:**
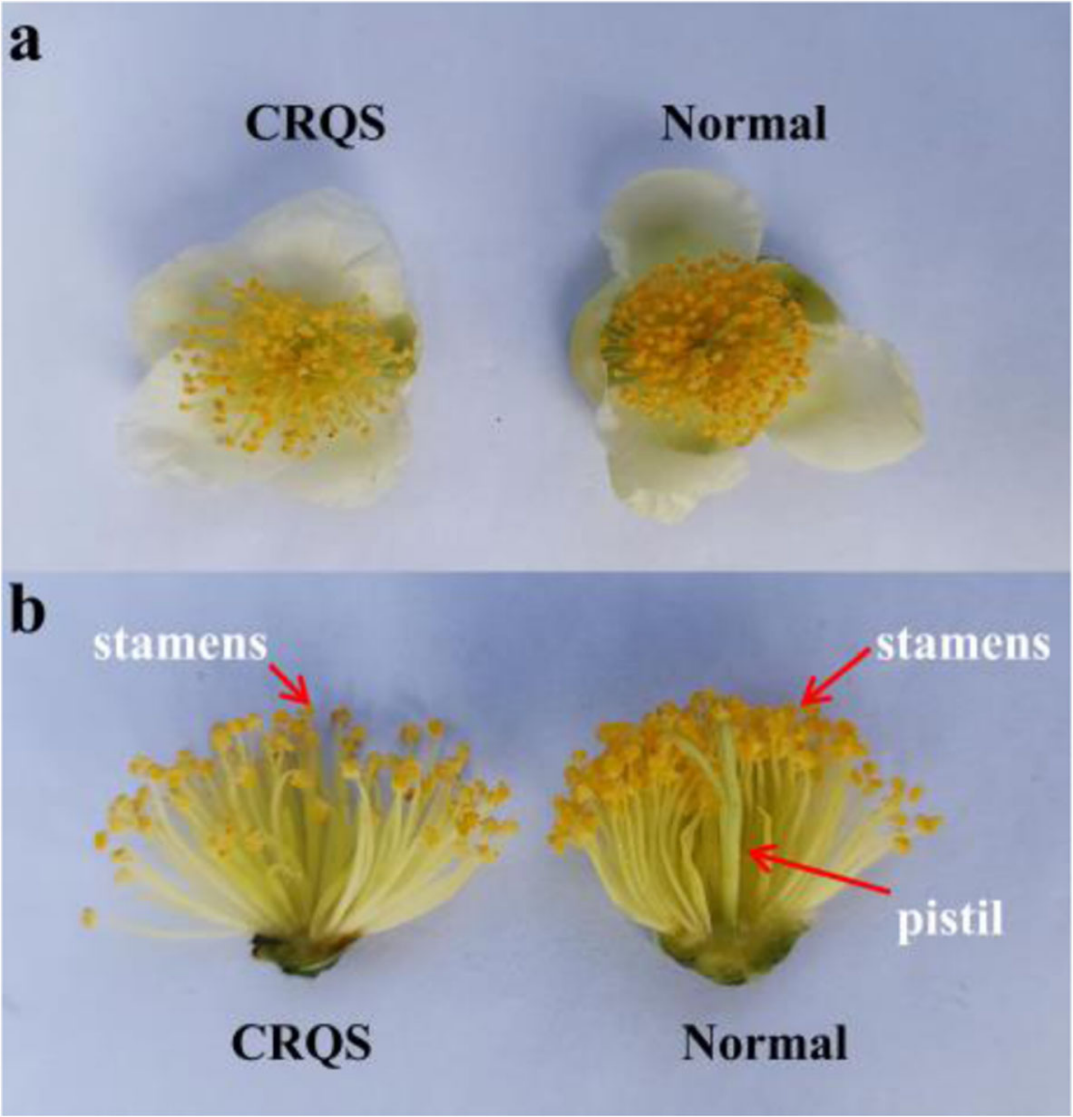
Morphological characteristics of the tea plant flower. (a) Morphologies of CRQS (pistil-deficient) and normal (bisexual) flowers. (b) Morphologies of the CRQS and normal floral organ

Flowers are the reproductive organs of the tea plant. The tea plant floral organ is a complete bisexual (perfect) flower composed of a pistil, stamens, petals, and calyxes. Our research group discovered a specific tea germplasm (CQRS) in the national tea germplasm repository of large-leaf tea (Menghai), which lacked the pistil and formed unisexual flowers (male flower; Fig. 1a). Except for the absence of pistils (stigma, style, and ovary), no significant differences in the stamens, morphology, and pollen fertility were observed between the pistil-deficient and normal flower buds (Fig. 1b).

### Sequencing and de novo assembly of transcriptome data

The total RNA from three tea plant flower samples was used for RNA-Seq with three replicates of each individual. After low-quality reads and adapter sequences were removed, the three cDNA libraries were subjected to pair-end reading on the Illumina HiSeq 2500 platform. Therefore, 58,150,274 clean reads remained (Table 2). The GC content of the clean data was above 43%, and the quality score (Q30) percentage was above 92% (Table 2). The ratios of mapped reads were 82.83–83.22% (CRQS), 79.98–80.48% (WT), and 82.34–82.96% (CT; Table 2). A total of 51,071 genes were obtained through comparison and analysis with the genome, among which 14,120 were new genes (Table S1).

**Table 2:** Summary dataset of transcriptome assembly

Sample Name		Clean Reads	Q30(%)	GC Content(%)	Total Mapped(%)
CRQS	CRQS_1	63,389,494	92.24	43.91	82.83
	CRQS_2	50,969,160	94.52	43.73	82.89
	CRQS_3	59,270,544	94.22	43.89	83.22
WT	WT_1	59,682,438	93.85	43.99	80.48
	WT_2	61,456,326	93.81	43.82	80.19
	WT_3	57,365,284	94.30	43.80	79.98
CT	CT_1	50,329,310	94.36	43.90	82.96
	CT_2	57,697,788	94.09	43.72	82.87
	CT_3	63,192,122	94.35	43.58	82.34

### DEGs and functional characterization

**Fig. 2:**
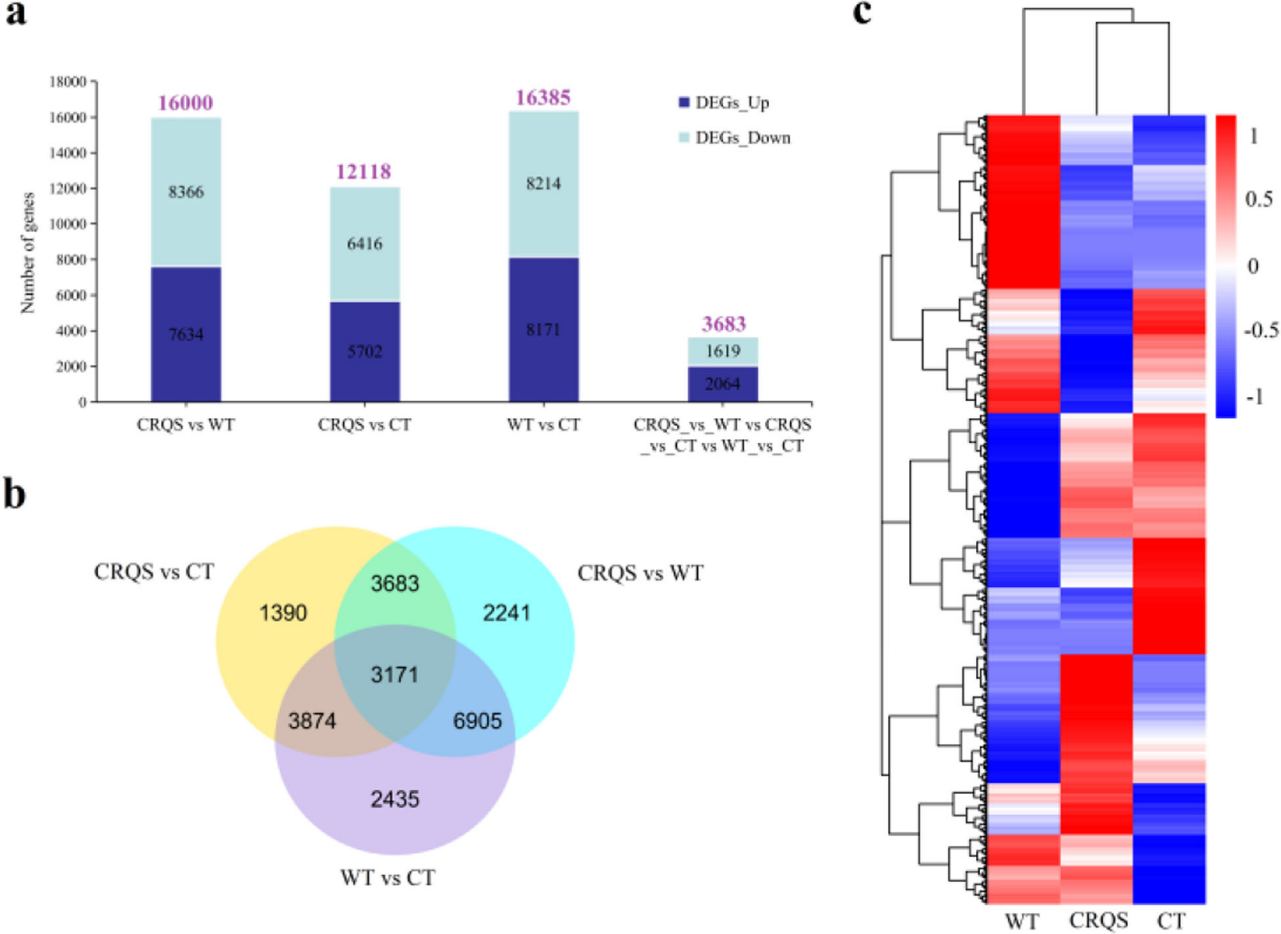
Cluster analysis of DEGs. (a) The numbers of downregulated and upregulated DEGs. (b) A Venn diagram illustrating the number of DEGs in CRQS vs WT, CRQS vs CT, and WT vs CT. (c) Heat map demonstrating the expression of the DEGs. High-expression genes are denoted in red, whereas low expression genes are denoted in blue on the heat map. The change in expression levels is illustrated after log10 conversion

To understand the difference in gene expression between pistil-deficient flowers (unisexual/male) and bisexual flowers, we measured the level of transcription of FPKM from RNA-Seq data. Genes were categorized as DEGs if they exhibited at least a 2-fold change (|log2 (fold change)| ≥ 1) in transcript abundance (*p* < 0.05) by using R package DESeq2. A total of 44,503 DEGs were detected in CRQS (Fig. 2), WT, and CT. Between CRQS and WT, 16,000 DEGs were screened out (Fig. 2a, b). The number of DEGs was 12,118 between CRQS and CT and 16,385 between WT and CT. In total, 3683 DEGs (Table S2) that differed from WT and CT were screened out in CRQS, with upregulated and downregulated expressions in 2064 and 1619 genes, respectively (Fig. 2a, b).

**Fig. 3:**
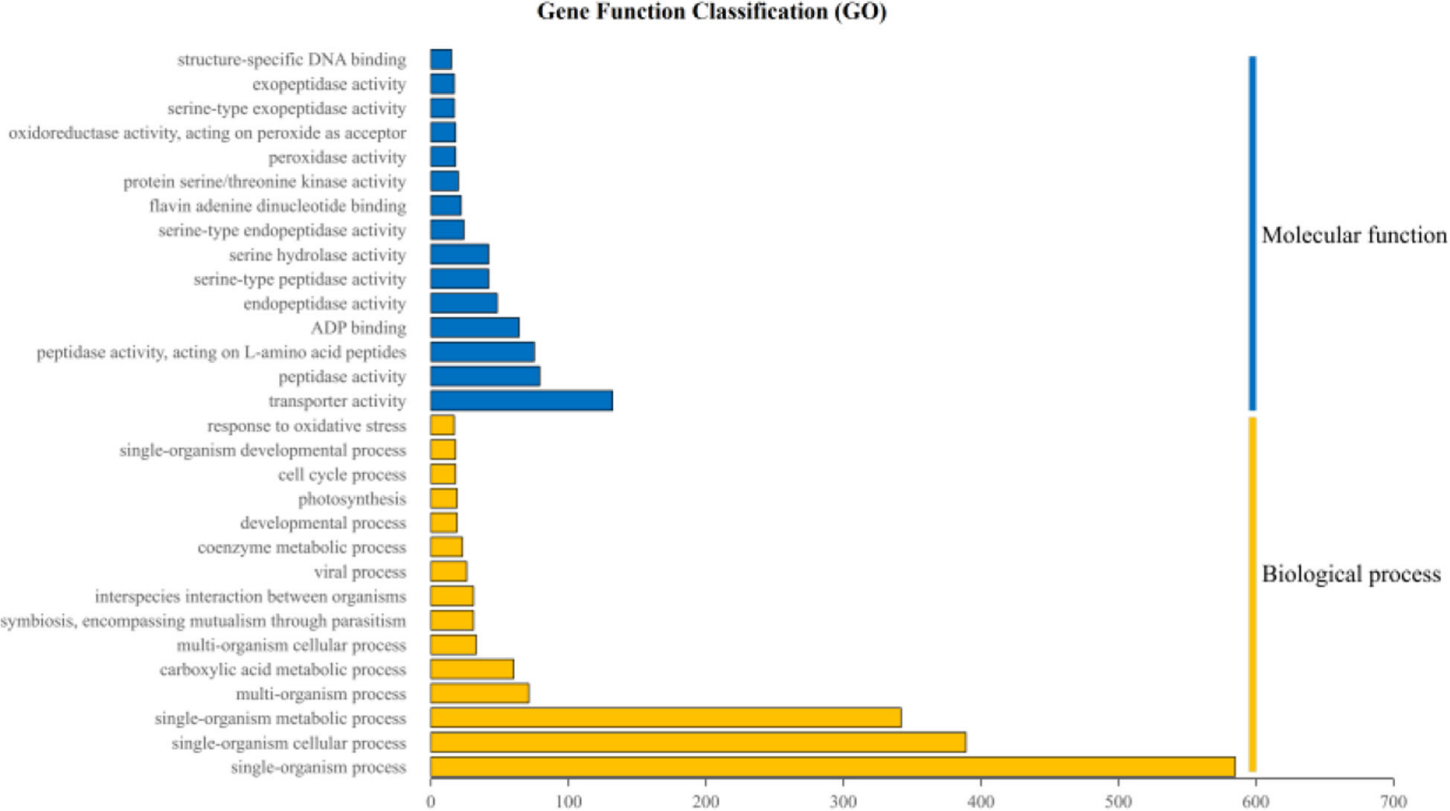
GO annotation classification statistics of the differential expression transcripts. Blue indicates molecular function, and yellow indicates biological process. According to the p-value from small to large, the first 30 components are displayed

**Fig. 4:**
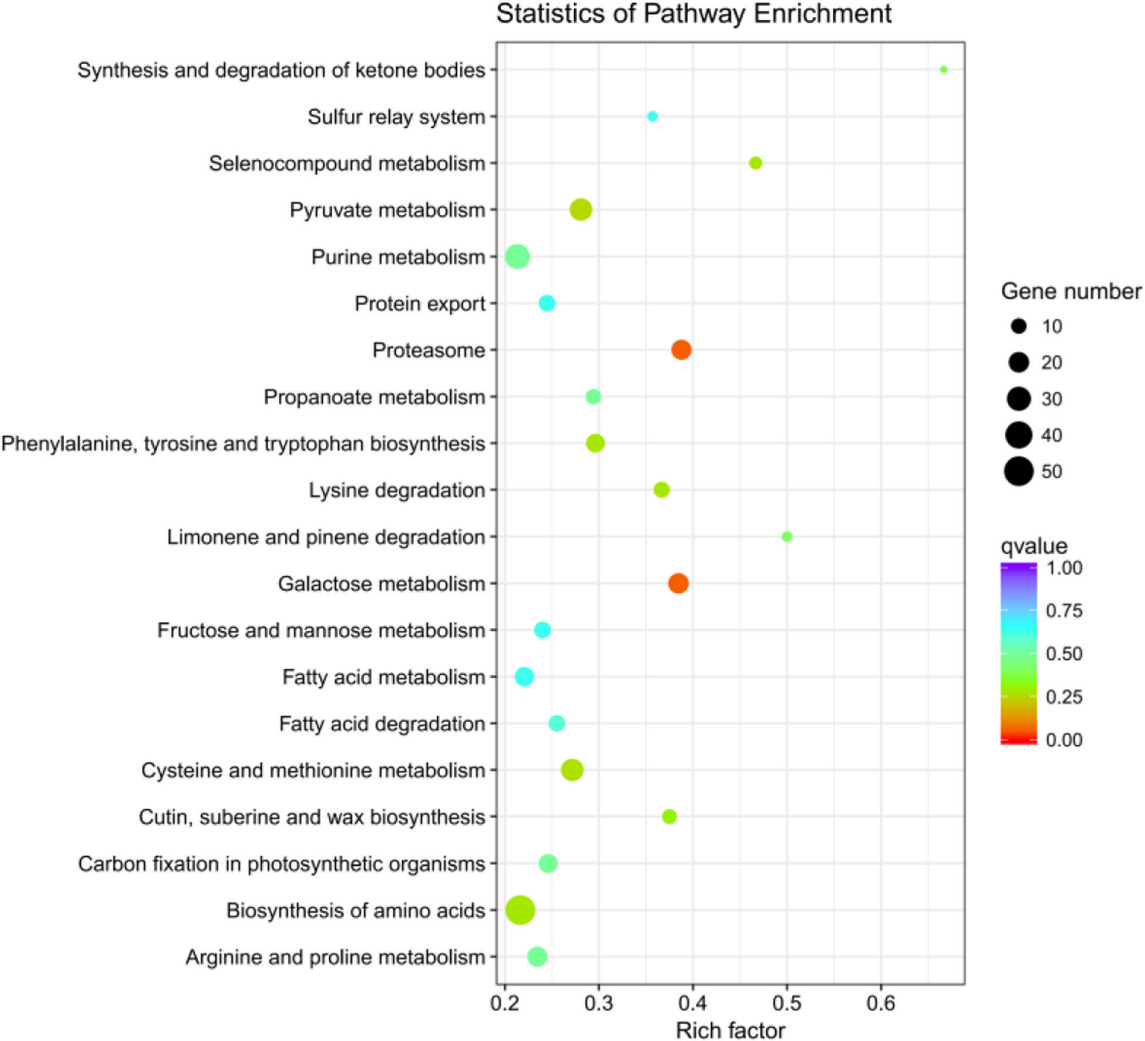
KEGG pathway classification statistics of the differential expression transcripts. According to the *p*-value from small to large, the first 20 components are displayed

The unique DEGs were characterized using the GO and KEGG databases. The major distribution of GO enrichment analysis was listed in Fig. 3 based on the Blast2GO software analysis. In total, 1825 genes were successfully annotated into 2285 terms within three main categories: cellular component (284), molecular function (719), and biological process (1282). The membrane, cell part, and cell accounted for a major proportion of the cellular component category. Binding and catalytic activity were the predominant genetic functions of the molecular function category. Most genes in the biological process category were involved in the metabolic and cellular processes. Moreover, the “hormone metabolic process”, “response to the hormone”, “regulation of hormone levels”, “reproduction”, and “reproductive process” were enriched in 2, 3, 2, 14, and 17 unigenes, respectively. The distribution of the KEGG pathway annotations for differential genes is illustrated in Fig. 4. A total of 118 KEGG pathways were identified and the majority were “galactose metabolism”, “proteasome”, “pyruvate metabolism”, “biosynthesis of secondary metabolites”, “biosynthesis of amino acids”, and “plant–pathogen interaction”.

### Identification of ABCDE model related genes involved in DEGs

The ABCDE model is widely accepted for explaining the development of floral organs. In this model, all genes belong to the MADS-box transcription factor family except the AP2 gene. Three AP2 (*CSA009488*, *Novel05812,* and *Novel10689*) and five MADS-box (*CSA016398*, *Novel01833*, *Novel05521*, *CSA014619,* and *CSA003190*) genes were screened in 3683 DEGs (Table 3). Within the AP2 genes, *CSA009488* exhibited a downregulated expression, whereas *Novel05812* and Novel10689 genes exhibited an upregulated expression. Annotation information and comparison results revealed that *CSA016398* (*AGL29*) and *Novel01833* (*AGL62*) belonged to the Mα Group, *Novel05521* (*AGL65*) belonged to the Mδ Group, and *CSA014619* (*CAULIFLOWER A-like*, *CAL*) and *CSA003190* (*AGL11*, *STK*) belonged to the MIKC group. Notably, only *CSA003190* was downregulated, and the other four AGAMOUS-like genes (*CSA014619*, *CSA016398*, *Novel01833*, and *Novel05521*) were upregulated (Table 3).

**Table 3:** DEGs related to tea flowers with pistil deletion

Gene Name		Gene ID	log2 (fold change)		
			CRQS/CT	CRQS/WT	Gene Expression Model
ACS	ACS3-like	CSA026751	7.2531	3.1576	UP
	ACS12-like	Novel05490	Inf	Inf	UP
ACO	ACO11-like	Novel11742	−1.1664	−1.0744	DOWN
	ACO6	CSA010549	2.621	3.8761	UP
	ACO4-like	Novel02945	−4.2473	−3.8215	DOWN
	ACO11	Novel10788	−8.0488	−7.8089	DOWN
ERF	ERF118-like	Novel00816	4.6473	3.9518	UP
WIP	WIP3	CSA026801	1.1716	1.6214	UP
AP2	AP2-like	CSA009488	−1.207	−1.1156	DOWN
	RAP2–7	Novel05812	Inf	10.465	UP
	RAP2–7	Novel10689	Inf	Inf	UP
AGL	CAULIFLOWER A-like (MIKC Group)	CSA014619	1.1677	1.1901	UP
	AGL65 (Mδ Group)	Novel05521	4.7555	5.2182	UP
	AGL11(STK, MIKC Group)	CSA003190	−2.839	−1.9205	DOWN
	AGL29(Mα Group)	CSA016398	6.4829	3.4336	UP
	AGL62(Mα Group)	Novel01833	Inf	Inf	UP

Note: Inf, infinity. UP indicates that the gene expression in CRQS was higher than that in WT and CT; DOWN denotes the opposite

### Identification of ethylene-related genes involved in DEGs

Studies have reported that ethylene is involved in the sex determination of various plants. We identified multiple genes related to ethylene synthesis, regulation, and signaling, such as two ACC synthetases (*CSA026751* and *Novel05490*), four ACOs (*Novel02945*, *Novel10788*, *CSA010549,* and *Novel11742*), one ERF transcription factor (*Novel00816*), and one WIP family transcription factor (*CSA026801*), by analyzing the DEGs (Table 3). The RNA-Seq results revealed that ACS genes were upregulated, whereas ACO genes were downregulated, except for *CSA010549*, which was upregulated. The transcription factor genes *Novel00816* and *CSA026801* were both upregulated (Table 3).

### qPCR analysis of DEGs

**Fig. 5:**
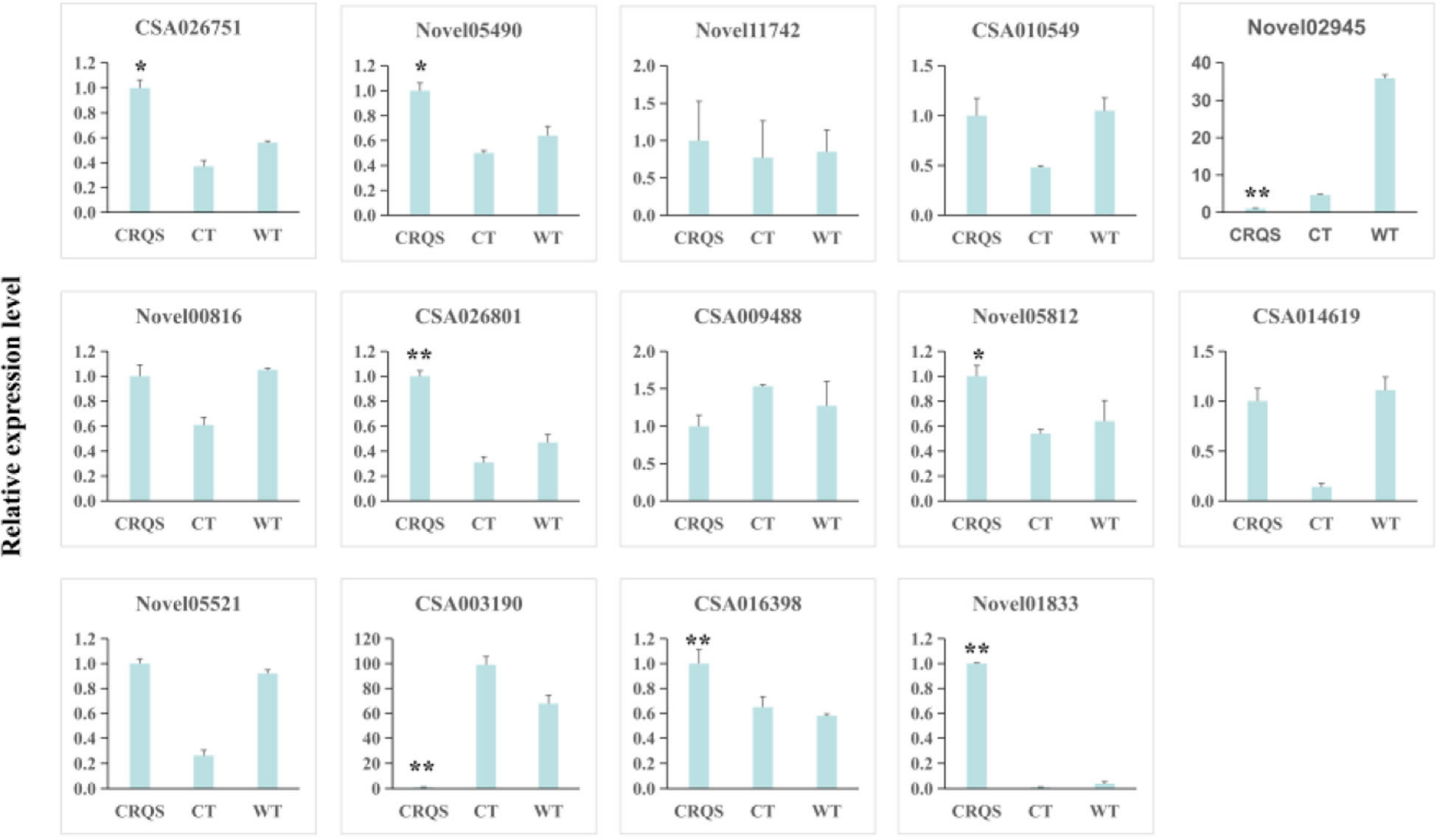
qPCR analysis of selected DEGs. Data are presented as means ± SD, *n* = 3 independent experiments. * *P* < 0.05; ** *P* < 0.01 (CRQS was compared with WT and CT)

To confirm RNA-Seq data, we designed primers for the 16 identified genes and performed qRT-PCR analysis (Fig. 5). The primers were designed multiple times based on the gene sequences of Novel10788 and Novel10689; however, their expression levels remained undetected. The qRT-PCR results indicated that the expression trends of most DEGs were consistent with transcriptome data (85.71%), except for the findings regarding *Novel11742* and *CSA010549* (14.29%). Thus, the credibility and reliability of RNA-Seq data were corroborated.

## Discussion

The tender shoots are the most economically valuable organs of the tea plant; however, during the 1.5-year reproductive growth period, the plant consumes large amounts of nutrients, which leads to a reduction in tea yield and quality and acceleration of plant aging, which in turn leads to a reduced economic life [3, 4]. The formation of unisexual or sterile flowers in tea plants effectively weakens the plant’s reproductive growth, which can make the plant more economically valuable. A natural unisexual tea plant mutant with no pistil was identified in the national germplasm repository of large-leaf tea (Menghai) [37]. This tea plant could not bear fruit because of the lack of pistil, and thus the reproductive growth was limited. The limited reproductive growth engendered the accumulation of nutrients, which promoted the growth of buds and leaves. The study of pistil-deficient tea plants clarified the mechanism of pistil-deficient and unisexual flower formation, which could enable the cultivation of nonfruitful tea plants and improve tea plant varieties. We studied the differences in the transcriptional expression profiles of pistil-deficient, cultivated, and wild tea plant flowers. A total of 3683 genes were screened from the three cDNA libraries, which are the different expressions in pistil-deficient tea plant flowers (Fig. 2). Moreover, enriched genes in 2285 GO terms and 118 KEGG pathways demonstrated the complexity of DEG function (Figs. 3 and 4).

### Role of ABCDE model–related genes expressed in pistil-deficient tea plant flowers

The ABCDE model can be used to further clarify the molecular mechanism of plant flower organ development [14–18]. Except for the A-class functional gene AP2, all genes related to the ABCDE model belong to the MIKC group of the MADS-box family. In *Arabidopsis*, the MADS-box family can be divided into five groups (Mα, Mβ, Mγ, Mδ, and MIKC) in addition to AGL33 [14, 16, 19, 42]. Therefore, the genes from the MIKC group play a crucial role in regulating plant flowering time, flower organ formation, and vegetative growth [42]. In this study, five MADS-box genes were identified from DEGs; among them, *CSA014619* (*CAULIFLOWER A-like*, *CAL*) and *CSA003190* (*AGL11*, *STK*) belonged to the MIKC group, and the other three genes belonged to other groups (Tables 3 and 4).

Among the DEGs belonging to the MICK group, *CAL* was primarily involved in the formation of the flower meristems and is not reported to be involved in pistil development. Studies have demonstrated that C, D, and E functional genes determine ovule development [14–17]. Furthermore, *CSA014619* was upregulated in transcriptome data but the qRT-PCR analysis results did not reveal a significant difference, which indicates that *CAL* may not be involved in pistil deletion in CRQS. CRQS varieties cannot develop ovules because of the lack of ovaries. *CSA003190* (*STK*) belongs to class D of functional genes. The transcriptome data and qRT-PCR results indicated that *CSA003190* in the pistil-deficient tea plant was extremely downregulated (Tables 3 and 4), which may be the reason why ovules were not form. This finding indicates that *CSA003190* may be involved in pistil deletion in CRQS.

The ABCDE model indicates that 2C + 2E quartet proteins determine carpel development [14]. In this study, no C and E functional genes were screened from DEGs. However, three A-type genes (*CSA009488*, *Novel05812*, and *Novel10689*) were identified. Studies have indicated that functional genes of classes A and C are antagonistic in action [43]. We determined that *Novel05812* was significantly upregulated (Tables 3 and 4). No changes in functional genes of class C were detected from the transcriptome data despite the upregulation of the class A functional gene *Novel05812*. The upregulation of class A functional genes may affect the formation of tea plant pistils in other manners.

### Role of ethylene-related genes in flower formation in pistil-deficient tea plant

Studies have demonstrated that ethylene affects pistil development in plants [23, 31, 32]. High concentrations of ethylene are conducive to the formation of female flowers, whereas low concentrations result in the formation of male flowers. The biosynthesis of ethylene involves two key enzymes, ACS and ACO. ACS catalyzes the conversion of SAM to 1-aminocyclopropane-1-carboxylic acid ester (ACC), and ACC is oxidized by ACO to form ethylene [27–29]. Numerous ACSs and ACOs are closely related to female flower formation in cucumbers and melons [30–35]. In this study, two ACS and four ACO genes were screened from DEGs (Tables 3 and Fig. 5). The two ACS genes were significantly upregulated as evidenced by both the transcriptome and quantitative results, but the downstream ACO gene was not upregulated. Notably, *Novel02945* (*ACO4-like*) was significantly downregulated.

Studies have indicated that CsWIP1 can directly bind to the promoter of *CsACO2* and inhibit its expression in cucumber plants, which affects the parthenocarpic composition [35]. Similar mechanisms have been observed in melons [34] and watermelons [36]. In the present study, the WIP transcription factor *CSA026801* was also identified in DEGs. Both transcriptome data and quantitative results indicated that *CSA02680* was significantly upregulated (Tables 3 and Fig. 5). These findings indicated that there may be a sex-regulating mechanism in tea plants similar to the one in cucumbers, whereby WIP inhibits the expression of ACO and forms unisexual flowers (male flowers). However, the ethylene signal response may differ from the conventional ethylene signal transmission.

## Conclusion

This study provides a comparative transcriptomic analysis of differences in gene expression in pistil-deficient and normal flower buds.

**Fig. 6:**
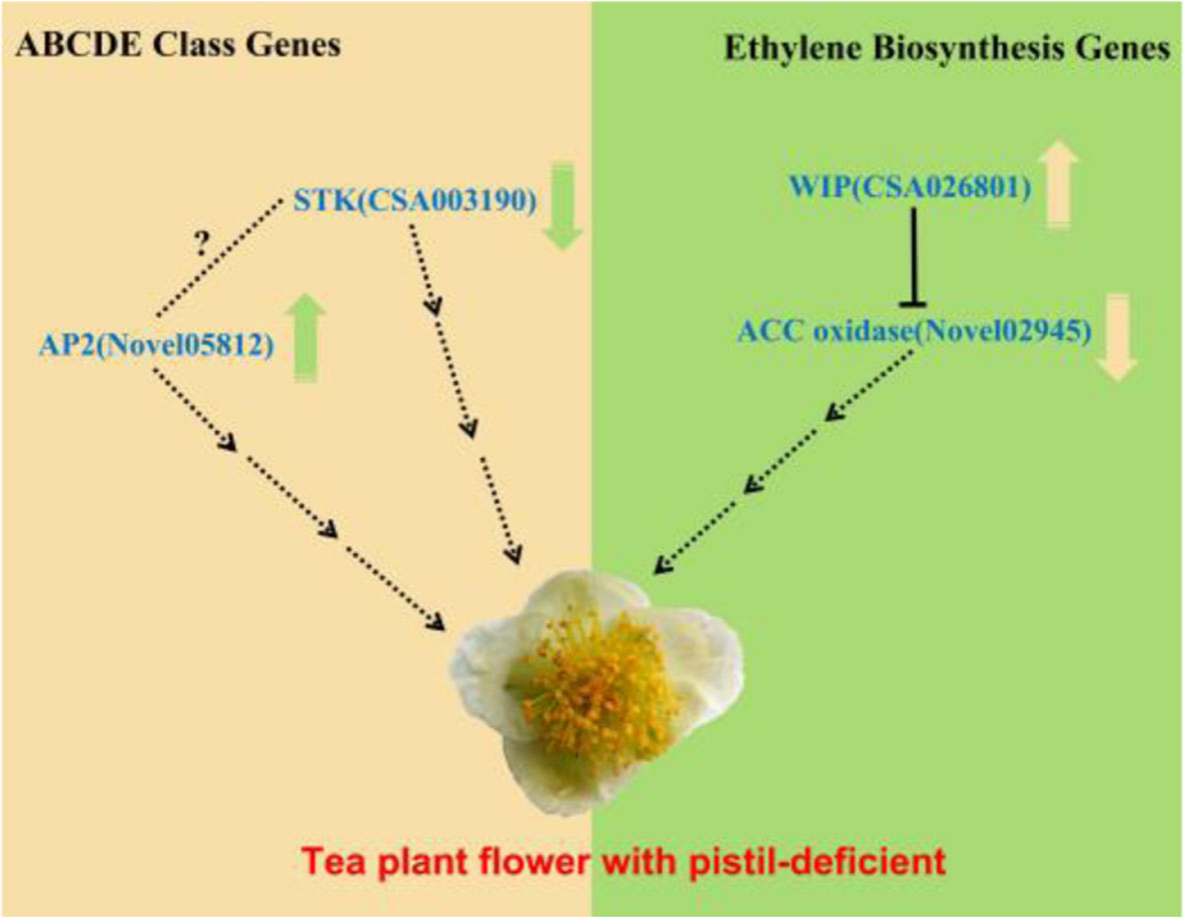
Putative gene regulatory network of pistil deletion in tea plants. The arrows (green or yellow) indicate the expression patterns of key genes in pistil-deficient tea flowers; upward arrows indicate upregulated expression and downward arrows indicate downregulated expression

A molecular model of pistil deletion (male flower) in the tea plant is summarized in Fig. 6. ABCDE-class functional genes A and D may be involved in pistil deletion, with a class A functional gene (*Novel05812*) being upregulated and a class D gene (*CSA003190*) being downregulated, thereby inhibiting or promoting other genes and inducing pistil deletion. Furthermore, *WIP3* (C*SA026801*) may inhibit the formation of the pistils in tea plants by downregulating the expression of *ACO4-like* (*Novel02945*) gene, leading to unisexual flowers. Overall, a large number of A-class, D-class, WIP transcription factors, and ACO genes are now identified as promising targets for further investigation and future breeding of sterile tea plant varieties.

## Supplementary information

**Additional file 1 :** **Table S1.** Basic annotation information for 14,120 new genes.

**Additional file 2 :** **Table S2.** Basic information of differentially expressed genes.

## Data Availability

We have provided detailed information about the materials and methods in our manuscript.

## Abbreviations

DEGs: Differentially expressed genes
GO: Gene Ontology
KEGG: Kyoto Encyclopedia of Genes and Genomes
CRQS: Pistil-deficient tea plan
WT: Wild tea plant
CT: Cultivated tea plant
ACS: ACC synthase
ACO: ACC oxidase
